# Novel and shared neoantigen for glioma T cell therapy derived from histone 3 variant H3.3 K27M mutation

**DOI:** 10.1186/2051-1426-3-S2-P445

**Published:** 2015-11-04

**Authors:** Yafei Hou, Gary Kohanbash, Kaori Okada, Shruti Shrivastav, Matthew Smith-Cohn, Theodore Nicolaides, Sabine Mueller, Angel Carcaboso, Ian Pollack, Hideho Okada

**Affiliations:** 1 University of California, San Francisco, San Francisco, CA, USA; 2 University of Utah, Salt Lake City, UT, USA; 3 Hospital Sant Joan de Déu Barcelona, Barcelona, Spain; 4 University of Pittsburgh, Pittsburgh, PA, USA

## Background and purpose

Malignant gliomas, such as glioblastoma (GBM) and diffuse intrinsic pontine gliomas (DIPG), are lethal brain tumors in both adults and children. Indeed, brain tumors are the leading cause of cancer-related mortality and morbidity in children. Children with DIPG have one-year progression-free survival rates below 25%, and median overall survival of 9 to 10 months with current treatment. Recent genetic studies have revealed that malignant gliomas in children and young adults often show shared missense mutations, which encodes the replication-independent histone 3 variant H3.3. Approximately 30 % of overall GBM and over 70% of DIPG cases harbor the amino-acid substitution from lysine (K) to methionine (M) at the position 27 of H3.3. The H3.3 K27M mutation in DIPG is universally associated with shorter survival compared with patients with non-mutated H3.3. We evaluated whether H3.3-derived peptides that encompass the H3.3 K27M mutation can induce specific cytotoxic T lymphocyte (CTL) responses in human leukocyte antigen (HLA)-A2^+^ CD8^+^ T cells.

## Methods

For prediction of HLA-A2-binding epitopes, an algorithm integrating peptide binding to HLA (NetMHC 3.4 server) and a proteosomal cleavage site prediction system (http://paproc.de/) was used. Four candidate peptides encompassing different amino-acid positions around the H3.3 K27M mutation were synthesized, and peptide-specific CTL lines and clones were generated from peripheral blood mononuclear cells of HLA-A2^+^ donors by in vitro stimulation with each of the synthetic peptides.

## Results

**Figure 1:**
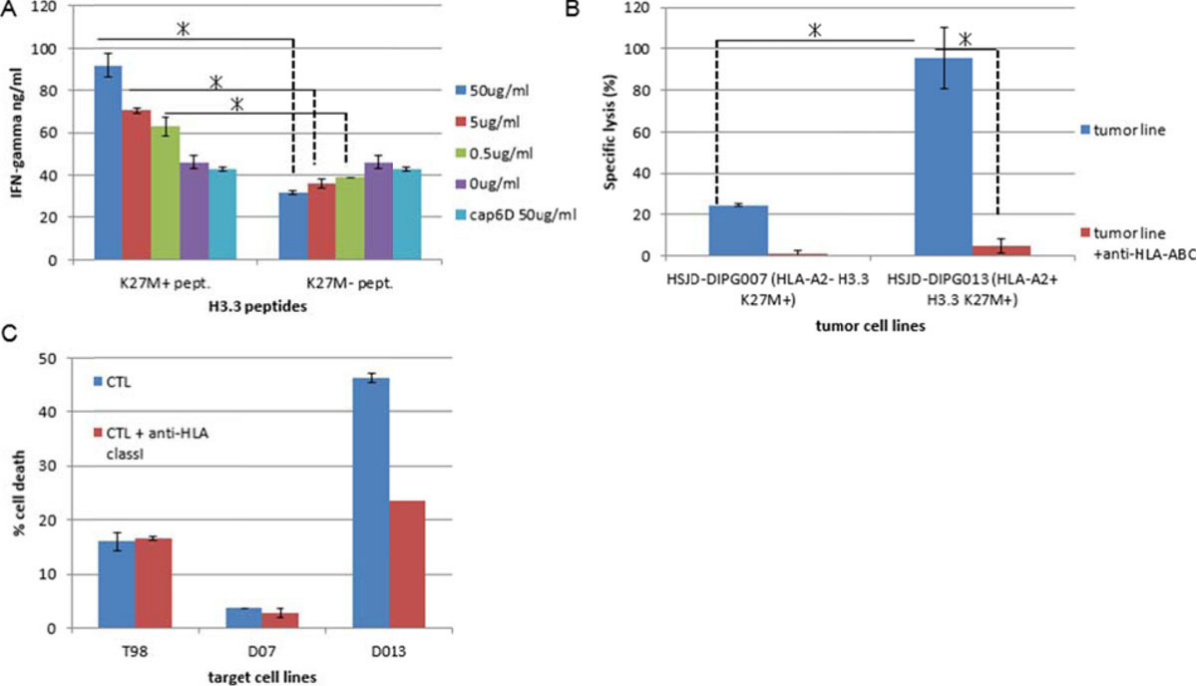


**Figure 2:**
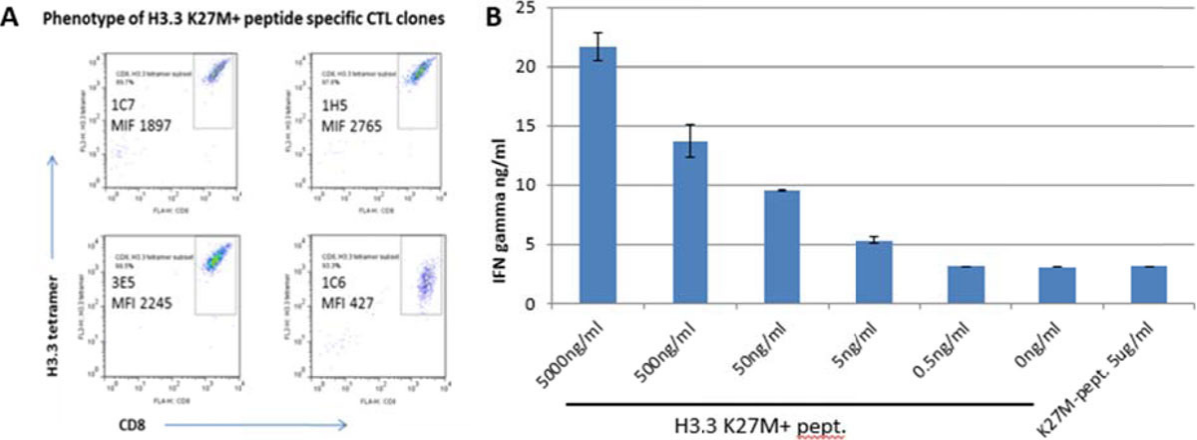


One of the 4 peptides (the H3.3.K27M epitope, hereafter) induced CTL lines which recognized not only the synthetic peptide loaded on T2 cells but also lysed HLA-A2^+^ DIPG cell lines which endogenously harbor the H3.3.K27M mutation. On the other hand, CTL lines did not react to HLA-A2^+^, H3.3 K27M mutation-negative cells or HLA-A2-negative, H3.3 K27M mutation^+^ cells (Figure 1). Furthermore, CTL clones with high and specific affinities to HLA-A2-H3.3.K27M-tetramer were successfully obtained (Figure 2), and α- and β-chain cDNAs from high-affinity T cell receptors (TCR)s were cloned into a lentiviral vector. Additional studies are underway to determine antigen specificity, key epitope residues in the epitope and possible cross-reactivity to naturally existing variants using T cells transduced with the lentiviral vector encoding the TCR. Assessments of *in vivo* immune responses to the epitope peptide and preclinical confirmation for absence of autoimmunity are also underway using HLA-A2-transgenic mice.

## Conclusions

These data provide us with a strong basis for developing peptide-based vaccines as well as adoptive transfer therapy using autologous T cells transduced with the TCR.

